# Gardner syndrome with giant abdominal desmoid tumor during pregnancy: a case report

**DOI:** 10.1186/s12893-020-00944-z

**Published:** 2020-11-12

**Authors:** Liquan Jin, Yunbo Tan, Ziting Su, Shan Huang, Sita Pokhrel, Hongbo Shi, Yiming Chen

**Affiliations:** 1 1St Department of General Surgery, The First Affiliated Hospital of Dali University, 32 Carlsberg Ave, Dali, 671000 Yunnan China; 2 Universal College of Medical Sciences, Bhairahawa, Nepal; 3 Institute of Translational Medicine for Metabolic Diseases, Dali University, Dali, Yunnan Province China

**Keywords:** Gardner syndrome, Desmoid tumor, FAP, Pregnancy, Case report

## Abstract

**Background:**

Gardner syndrome is a subtype of familial adenomatous polyposis (FAP), characterized by a combination of adenomatous intestinal polyps and extracolonic lesions such as multiple osteomas, dental abnormalities, and soft tissue tumors. Although 12% of patients with intestinal polyposis of FAP may occur intra-abdominal desmoid tumors, pregnancy complicating with giant abdominal desmoid tumors is a relatively rare case.

**Case presentation:**

A 28-year-old pregnant woman was diagnosed with Gardner syndrome in whom an intra-abdominal tumor was found a year after undergoing a laparoscopic total colectomy due to family adenomatous polyposis. At 32 weeks’ gestation, she presented to our department for the third time complaining upper abdominal pain caused by the giant abdominal mass about 21 × 12 cm^2^ in size. After multidisciplinary consultation and discussion, the decision of fetal preservation treatment was made. After the delivery of a baby girl, abdominal mass resection was performed, and pathological examination revealed a fibrous adenoma. The patient was discharged after a week and was uneventful in the follow-up for half a year.

**Conclusions:**

Gardner syndrome is characterized by typical syndrome including family adenomatous polyposis and extra-intestinal tissue tumor. Were desmoid tumors rarely as large as fetus and local aggressively. In our case, we selected surgery to remove the intra-abdominal desmoid tumor after the natural delivery of the fetus and no abnormalities were observed during the 6 months follow-up. Women during pregnancy have an increased risk for the development of desmoid tumors, likely with the sex hormone to be one of the triggers. Therefore, we suggested that when a patient with Gardner syndrome desire to conceive again, they should go to the hospital for a regular review at least once every 3 months.

## Background

Gardner syndrome, mainly manifested by multiple gastrointestinal polyps and universal lesions such as soft tissue tumor, ectopic teeth, osteoma, and retinal pigment epithelium, is a rare autosomal dominant genetic disorder caused by gene mutation in adenomatous polyposis coli (APC). Occasionally, Gardner syndromes also known as "deep" or "invasive" fibromatosis, characterized by fibroblastomas or desmoid tumors that originated from deep muscle or aponeurosis.

The World Health Organization (WHO) classified desmoid tumors as one of the moderate fibroblastoma, which recurs locally and invasively rather than distant metastasis. The incidence of Gardner syndrome is 2–4 per million globally, more in females. Moreover, a few factors were identified to be responsible for desmoid tumor: surgery history, trauma, oral contraceptives, reproductive age and delivery history. To diagnose the desmoid tumor, spindle cells in the dense collagen stroma while rare mitosis and necrosis could be found under histopathological examination. In this paper, we report a case of Gardner syndrome in a female with a giant abdominal desmoid tumor during pregnancy.

## Case presentation

**Fig. 1:**
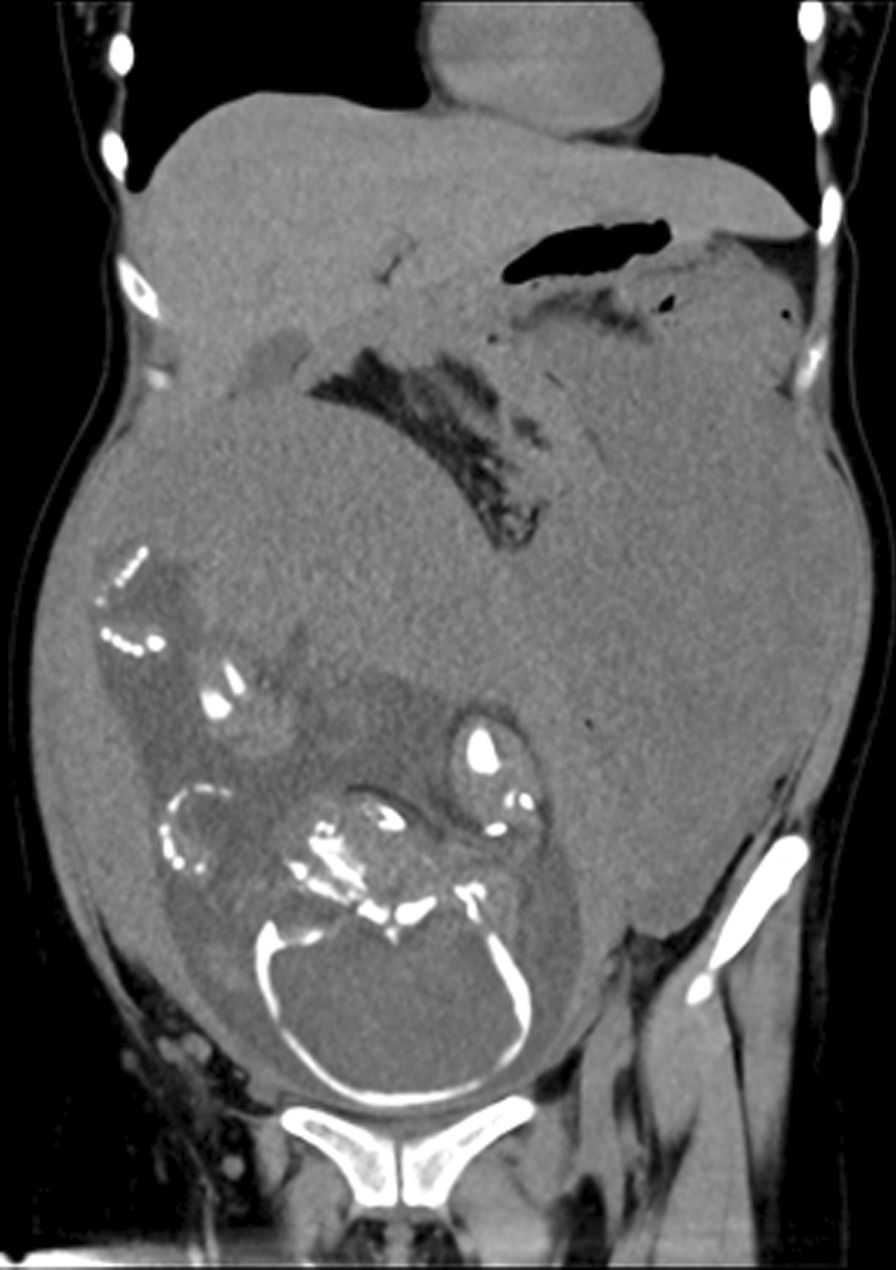
CT scanning showed a huge occupant in left side, abdominal cavity and the fetal in the right with 31 weeks

**Fig. 2:**
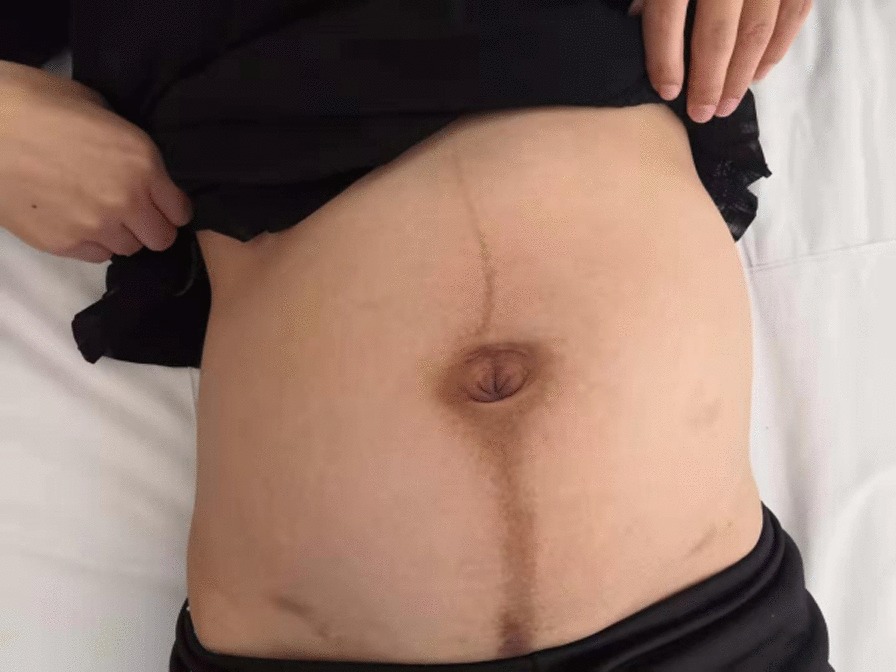
One month after delivery, the abdomen is visible as pregnant

**Fig. 3:**
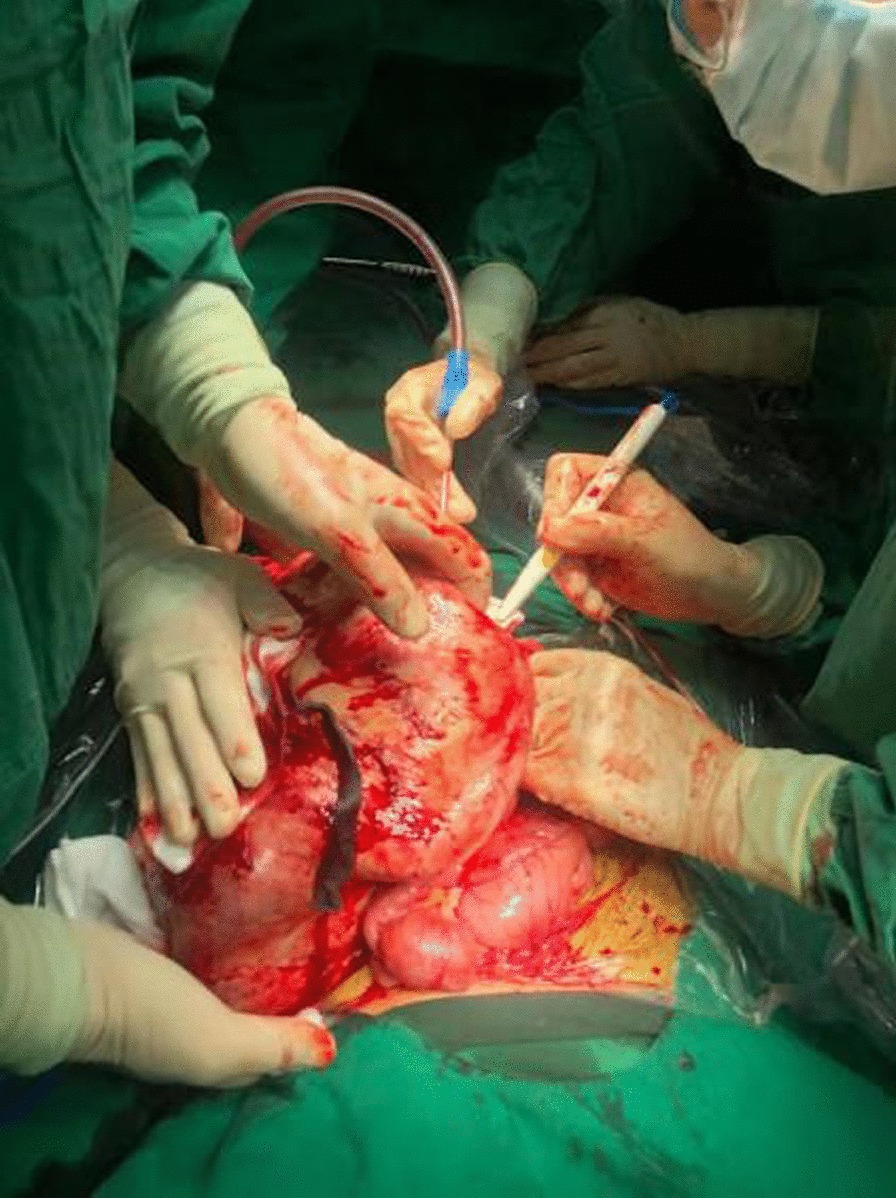
Surgery section the huge abdominal mass

**Fig. 4:**
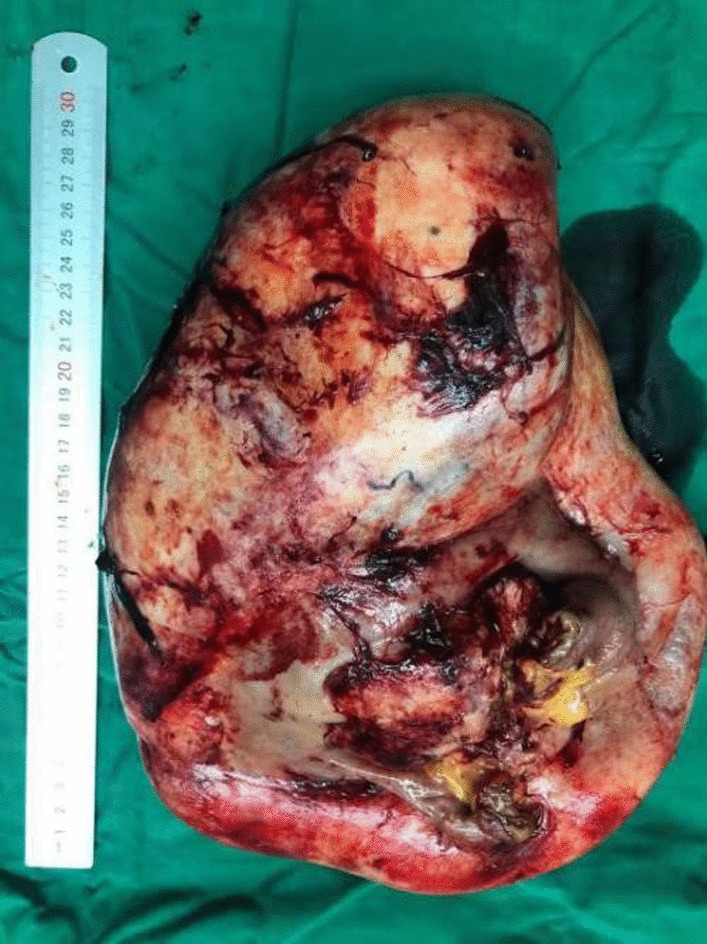
Removal of specimen (30 cm*15 cm*5 cm)

**Fig. 5:**
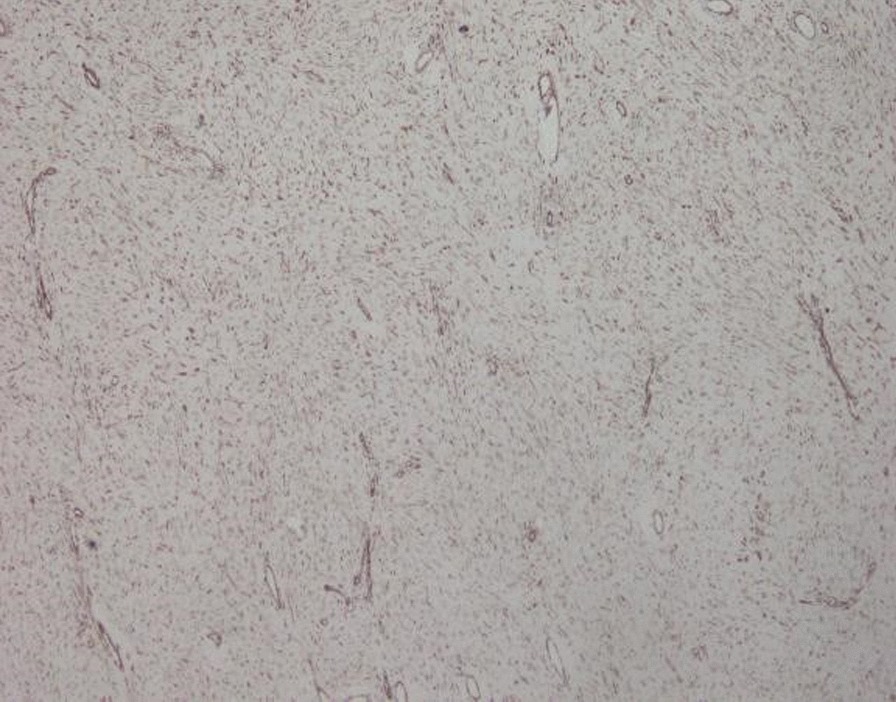
Postoperative pathological examination showed ligament-like fibrous adenoma

A 28-year-old female, gravida 2, para 1, discovered a massive mass in the abdominal cavity during the 8th month of pregnancy. Three years ago, this patient was admitted to our hospital in the Hematology and Oncology Department due to "repeated dizziness, fatigue and shortness of breath after activity". She was admitted at 60 g/L hemoglobin and was transfused with 3.0U of the same type of red blood cell suspension. The microscopic examination of colon revealed extensive polyp-like lesions in the entire colon. On-duty doctor in responsible for this case suspected "familial adenomatous polyposis (FAP)" and suggested transfer of this patient to our surgical department for further evaluation and treatment. After completing the preoperative examination and investigation, a laparoscopic total colectomy was performed. Postoperative pathological examination showed tubular adenomas (low-grade) and more than 1000 polyps, most of which were broad-based and fit the family adenomatous polyposis. One year later, fibroid adenoma was found in the upper right pubic symphysis. Taking the intestinal lesions into consideration, this case was diagnosed as "Gardner syndrome". In August 2019, the patient presented for the third time to our hospital due to upper abdominal pain at 32 weeks of pregnancy. A CT examination showed that the left side of the uterus in the abdominal cavity was occupied by a huge mass about 21 × 12 cm in size, with mild hydronephrosis in both kidneys (Fig. 1). The patient was diagnosed with abdominal mass with intrauterine pregnancy at 31 weeks with bilateral hydronephrosis. After multidisciplinary consultation and discussion at our hospital, the decision of fetal preservation treatment was made. In October 2019, 1 month after the delivery of a baby girl, the patient still looked like as pregnant according to the abdominal appearance (Fig. 2). An ‘Extreme mass Abdominal Mass Resection’ was performed at our department (Figs. 3, 4). The postoperative pathological examination showed that the ligament-like fibrous adenoma (Fig. 5), combined with the medical history, met the diagnosis criteria of Gardner Syndrome. The patient was discharged after one week and no abnormality was found in the follow-up for half a year.

## Discussion and conclusion

Gardner syndrome is characterized not only by typical syndrome including family adenomatous polyposis and extra-intestinal tissue tumor but also by a rare presentation with giant abdominal desmoid tumors during pregnancy [1]. The desmoid tumor can be as large as a fetus and the tumor can be locally aggressive, so it is hard to decide for the available treatment options. In our case, although the celiac desmoid tumor was huge, the fetus was in good condition without intrauterine distress or hypoxia and the fetus development was normal. Therefore, after the natural delivery of the fetus, an operation to remove the desmoid tumor is an appropriate treatment option to avoid fetal dyspnea, dysplasia, and increased maternal mortality. Early operation to prevent local desmoid infiltration and mesenteric desmoid tumors, which is the second leading cause of mortality in FAP patients [2], would have increased the maternal mortality risk. The localization of desmoid tumors is generally classified as intra-abdominal in the abdominal wall or extra-abdominal [3]. Desmoid tumors most commonly involve the extra-abdominal locations in the general population, whereas patients with FAP mostly present with intraabdominal disease. Only 5% of sporadic desmoid tumors are intra-abdominal, but 80% of patients with familial adenomatous polyposis (FAP)-associated desmoid tumors develop intra-abdominal disease [4]. The incidence is 3% for soft tissue sarcomas and about 0.03% for all malignancies [5].

Gardner's syndrome is associated with familial adenomatous polyposis (FAP), involving a mutation in anaphase promoting complex gene and several extra-digestive manifestations: osteomas, epidermal cysts and desmoid tumors [6]. Approximately 7.5% of desmoid tumors are associated with familial adenomatous polyposis (FAP) in the general population. There is a special relationship between desmoids and FAP (Gardner syndrome), with an incidence of 3.5–32% [7]. The usual presentation is a slowly growing mass without associated pain or discomfort. Depending on the location of the tumor, it may present with symptoms such as neurological deficit, joint stiffness or abdominal complaints. In our case, the patient complained of abdominal pain during pregnancy. Despite the significant size of the mass, the abdominal pain was not so severe, hence, the patient presented for delayed consultation. As a result, the fetal growth during pregnancy can be overlooked with the presence of abdominal masses. Moreover, failure to recognize Gardner syndrome combined with familial intestinal polyps resulted in the growth of desmoid tumors in the abdominal cavity. Therefore, we suggested that when a patient with an age of below 30 with FAP and the history of extra-abdominal desmoid tumors, the possibility of intraperitoneal desmoid tumor growth should be taken into consideration. If the patient desire to conceive again, they should go to the hospital for a regular review at least once every 3 months.

Although the mechanism of desmoid tumors remains largely unknown, desmoid tumors might be driven by alterations of the Wnt/APC/β-catenin pathway [8], e.g., sporadic desmoid tumors are associated with somatic mutations of CTNNB1, and germline mutations of APC and somatic mutations of CTNNB1 are probably mutually exclusive. The rate of cases diagnosed with core-needle biopsies and CTNNB1 mutational analysis increased from 30.6 to 40.7% and from 87.8 to 94.1%, respectively. The mean delayed for pathological diagnosis confirmation constantly decreased from 107 to 47 days [9]. In addition, hormonal, genetic and physical factors all play a role in the development and growth of desmoid tumors. Desmoids occur between the age of 15 and 60 years, but particularly during early adolescence and with a peak age of about 30 years. Women during pregnancy have an increased risk for the development of desmoid tumors [3, 10, 11], likely with the sex hormone to be one of the triggers.

Regarding the treatment of desmoid tumors, new alternatives emerged especially in primary non-resectable locations in recent years. Initially, local surgery is the first chosen treatment for desmoid tumors. With advanced techniques, large *en bloc* surgery is no longer regarded as a cornerstone treatment for desmoid tumors, given that the relapse rate after surgery exceeds 60% in larger series, and that spontaneous regression is documented to be approximately 25% of the cases [12]. Therefore, there is a current shift to a more conservative approach, namely the ‘wait-and-see policy’ [13], which is currently recommended as the first approach in Desmoid-type fibromatosis (DTF) [14]. However, a nationwide prospective cohort [15] showed that there was no difference between patients undergoing an operation and those managed by the wait-and-see policy in terms of two years of event-free survival (EFS). Among the patients with favorable locations (abdominal wall, breast, intra-abdominal and lower limb), the 2-year EFS was similar in patients treated by either surgery or the wait-and-see approach. Among patients with unfavorable locations (chest wall, head and neck and upper limb), the 2-year EFS was significantly enhanced in patients initially managed with the wait-and-see approach compared with those who underwent initial surgery. Nevertheless, systematic therapy is an option in unresectable or recurrent diseases. Available options include hormonal therapies, non-steroidal anti-inflammatory drugs (NSAIDs), interferon, and chemotherapy. The use of hormonal therapy for the treatment of these tumors is based on the association of these tumors with pregnancy or contraceptives pills and reports of regression after menopause or oophorectomy. Success rates of around 50% have been obtained with hormonal treatments and other agents such as NSAIDs, Vitamin C and warfarin. The most common regimen uses high dose tamoxifen at 120 mg per day along with sulindac and chemotherapy (imatinib and doxorubicin) [16]. In our case, we selected surgery to remove the intra-abdominal desmoid tumor after the natural delivery of the fetus and no abnormalities were observed during the 6 months follow-up. Women during pregnancy have an increased risk for the development of desmoid tumors, likely with the sex hormone to be one of the triggers. Therefore, we suggested that when a patient with Gardner syndrome desire to conceive again, they should go to the hospital for a regular review at least once every 3 months.

## Data Availability

Not applicable.

## Abbreviations

FAP: Familial adenomatous polyposis
APC: Adenomatous polyposis coli
WHO: World Health Organization
CT: Computed tomography
CTNNB1: A protooncogene gene encodes for b-catenin
DTF: Desmoid-type fibromatosis
EFS: Event-free survival
NSAIDs: Non-steroidal anti-inflammatory drugs
